# Direct-Write Printing for Flexible and 3D Electronics: Aerosol Jet vs. Micro Dispensing

**DOI:** 10.3390/mi16080931

**Published:** 2025-08-13

**Authors:** Ankur Gohel, Mathieu Gratuze, Mohsen Ketabi, Ricardo Izquierdo

**Affiliations:** Laboratoire de Communication et d’Intégration de la Micro-Électronique (LaCIME), Regroupement Stratégique en Micro Systèmes du Québec (ReSMIQ), École de Technologie Supérieure, 1100 Notre Dame Ouest, Montreal, QC H3C 1K3, Canada; ankur.gohel@lacime.etsmtl.ca (A.G.); mathieu.gratuze@lacime.etsmtl.ca (M.G.); mohsen.ketabi@lacime.etsmtl.ca (M.K.)

**Keywords:** direct-writing technology, printed and flexible electronics, 3D printed electronics

## Abstract

This study provides a comprehensive comparison of two leading direct-write manufacturing technologies: Aerosol Jet Printing (AJP) and Micro Dispensing Technology (MDT). The investigation examines their capabilities, limitations, and performance characteristics for printing on both 2D and 3D substrates. The findings offer valuable insights into the suitability of each printing method for flexible electronics based on the morphology and electrical performance of the deposited inks. The results reveal distinct advantages for each technique: AJP excels in resolution, while nScrypt’s micro dispensing offers superior 3D conformality, greater material versatility, and higher throughput.

## 1. Introduction

The rapid advancement in electronic device miniaturization and increasing complexity have significantly challenged traditional manufacturing techniques, driving the need for advanced additive manufacturing technologies. Non-contact methods such as inkjet printing, aerosol jet printing, and material dispensing technology have emerged as crucial innovations, enabling precise deposition of functional materials with exceptional resolution and versatility [1]. Non-contact additive manufacturing technologies represent a paradigm shift from conventional subtractive and contact-based manufacturing processes [2]. These advanced techniques enable direct material deposition with minimal substrate interaction, reducing material waste and mechanical stress while providing exceptional geometric flexibility [2,3]. The capability to print on a wide range of substrate geometries, such as flexible, curved, and three-dimensional (3D) surfaces, is afforded by these methods.

These cutting-edge approaches are revolutionizing the fabrication of electronic circuits, sensors development, and multifunctional devices, meeting the demands of next-generation manufacturing [4].

**Figure 1:**
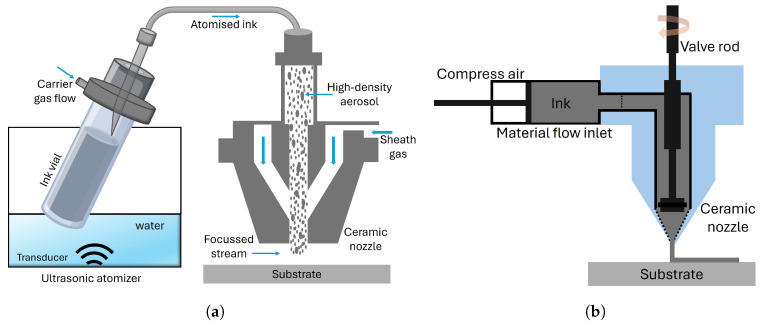
A schematic view of the two direct-writing technologies: (**a**) AJP and (**b**) MDT.

Commercial Aerosol Jet Printing (AJP) systems are primarily available from two manufacturers: Optomec© and the Integrated Deposition Solutions (IDS) NanoJet system [5]. While Optomec© offers comprehensive systems tailored for both research and industrial applications, IDS adopts a modular approach, allowing components such as the deposition head to be purchased separately. Both platforms employ ultrasonic atomization; however, their aerosol generation mechanisms differ. Optomec© systems use a separate ink vial placed in an external water-based ultrasonic bath, with the aerosol transported via PTFE (Polytetrafluoroethylene) tubing to the print head. In contrast, the IDS NanoJet^®^ system integrates the ink cartridge within the deposition head itself, where aerosol is generated directly at the point of use through membrane excitation by an ultrasonic transducer coupled via water. AJP fundamentally operates through the aerodynamic focusing of aerosolized ink particles, offering precise control in depositing functional materials, as shown in Figure 1 [6]. The process begins with the transformation of liquid inks into fine aerosol streams, typically through pneumatic or ultrasonic atomization. These aerosolized particles are then directed through a nozzle with the aid of a precisely controlled sheath gas, which focuses the stream onto the substrate surface. This aerodynamic focusing mechanism ensures high accuracy and control, allowing for the creation of intricate patterns with minimal overspray. AJP exhibits exceptional versatility in material compatibility, accommodating a wide range of viscosities from 1 to 1000 cP [7,8]. Furthermore, AJP can achieve feature resolutions as fine as 10–50 μm [6,9], making it ideal for applications demanding high precision, such as antenna fabrication [10], transistors [11], electronics interconnections [12], micro-sensors [13], and biomedical devices [14].

On the other hand, Micro Dispensing Technology (MDT) represents an alternative non-contact printing approach, utilizing a SmartPump and a valve system to deposit materials with exceptional accuracy and control, as shown in Figure 1 [15]. This technique supports an extensive viscosity range from 1 to 1,000,000 cP [16], enabling deposition of diverse materials, including conductive pastes, polymeric inks, and adhesives [17]. With minimal standoff distances (25–100 μm) and feature sizes approaching 20 μm, micro dispensing facilitates intricate electronic circuit fabrication and advanced multi-material manufacturing strategies [18]. This technology is considered a viable alternative to AJP.

Both contact-less technologies AJP and MDT offer enhanced flexibility, scalability, and variability compared to traditional contact-based technologies [17,19]. MDT has been widely reported as a deposition method for applications in printed electronics, including wearable sensors [20,21,22], antennas [23], solar cell front metallization [24], fuel cells [25], and hybrid electronic circuits [26]. Despite its potential, MDT has been less explored in the scientific literature compared to AJP, with a noticeably smaller number of publications. Studies encompassing both technologies remain limited. Zhang et al. [19] and Blackburn et al. [16] reviewed AJP and MDT as direct-writing technologies, presenting a comparative analysis of printing parameters. Rodriguez et al. [27] assessed the capabilities of MDT for fine-line printing on polyimide substrates. Schlake et al. [28] introduced a preliminary approach to combining AJP and MDT, aiming to synergize the characteristics of the two technologies without doing a comparison related to the laser sintering. In contrast, AJP has been studied more extensively. Ratnayake et al. [29] explored the physics underlying aerosol-jet-printed strain gauges, providing detailed insights into the deposition mechanics. Investigations into silver tracks deposited by AJP were carried out by Smith et al. [30] on different substrates, while Jeong et al. [31] examined the influence of process parameters on the geometry of aerosol jet printed silver lines. Further contributions by Mahajan et al. [6] and Goh et al. [32] analyzed the relationship between aerosol-jet-printed line widths and process parameters such as impact exhaust flow rate and atomizer gas rate.

Similar direct-write dispensing technologies are also accessible by Micropen Technologies (Exxelia SAS, Paris, France) and Voltera NOVA (Voltera Inc., Ontario, Canada), both recognized for their capabilities in high-resolution material deposition. These platforms have been utilized in advanced applications, including medical device fabrication and printed electronics. Zhao et al. [33] demonstrated the formulation of silver nanoparticle-based ink enhanced with graphene and copper for Micropen printing on paper substrates. Their work highlights the role of silver nanoparticles synthesized via sodium borohydride reduction as the primary conductive filler and how their integration with graphene or copper improves mechanical flexibility and electrochemical performance. Further, Cai et al. [34] presented the fabrication of platinum microheaters on alumina substrates using a Micropen for direct deposition of platinum paste followed by laser sintering. This method enables precise material patterning with reduced waste, yielding well-sintered platinum films with strong adhesion and low electrical resistivity. El-Hajj et al. [35] utilized the Voltera NOVA platform to fabricate silver-based biomedical tattoo electrodes via both inkjet and extrusion printing, enabling the creation of highly conductive, skin-conformal electrodes with low impedance and strong mechanical performance. Notably, extrusion-printed electrodes exhibited significantly lower sheet resistance and superior contact interface, highlighting the critical role of ink viscosity and substrate interaction in print quality.

This study presents a comprehensive comparison between two leading direct-write manufacturing technologies: AJP and MDT. The investigation focuses on their capabilities, limitations, and performance characteristics for printing across both 2D and 3D substrate. The findings offer insights into the suitability of each printing technique for flexible electronics based on the morphology and electrical performance of the deposited ink. The results demonstrate distinct advantages for each technology, with AJP showing superior resolution, while nScrypt’s micro dispensing exhibits advantages in 3D conformality, material versatility, and throughput.

## 2. Material and Methods

AJP was performed using direct-write aerosol jet printer (Aerosol Jet^®^ 300, Optomec Inc., Albuquerque, NM, USA). The printing parameters, including nozzle diameter, atomizer settings, and carrier gas flow rates, were optimized to achieve uniform and precise deposition on 2D and 3D substrates. The aerosol jet printing ultrasonic atomizer process was conducted using a nozzle with a 300 μm diameter at a printing speed of 5 mm/s. The sheath flow and atomizer flow rates were set to 50–60 sccm and 25–35 sccm, respectively, with an atomizer power of 45–48. A substrate stand-off distance of 4 mm was maintained, and the deposition rate was controlled at 0.1 mL/min. The substrate temperature was held constant at 60 °C to ensure optimal ink adhesion and feature uniformity.

For all the experiments using MDT, an nScrypt 3Dn-300 (nScrypt, Orlando, FL, USA) printer was used. The machine equipped with a pneumatic SmartPump™ system enables high-precision deposition of functional materials for advanced manufacturing processes. The micro dispensing process utilized a nozzle with an inner diameter of 50 μm and an outer diameter of 100 μm, ensuring fine resolution for material deposition. The valve was precisely controlled, with an opening position set to 3.190 mm and a closing position of 3.170 m>m, operating at a speed of 0.099 mm/s to regulate the material flow accurately.

A consistent dispensing gap of approximately 200 μm was maintained between the nozzle and the substrate, while the substrate temperature was controlled at 60 °C to optimize material viscosity and adhesion. The system operated under a dispensing pressure of 4 psi, with a printing speed of 40 mm/s, ensuring high precision and uniformity in the deposited patterns. Table 1 presents the printing parameters utilized in this morphological study for both AJP and MDT. These parameters were carefully optimized to achieve reliable and reproducible deposition of silver ink on both flat and 3D substrates.

For both printing processes, silver nanoparticle ink (Sicrys™ I30EG-1, PV Nano Cell Ltd., Migdal HaEmek, Israel) with a metal loading of 30 wt% was utilized. The ink contains Ethylene Glycol (EG) solvent and has particle sizes ranging from approximately 70 to 115 nm. It is specifically designed for ultrasonic atomization printing on substrates such as Polyimide (PI), Glass, Polycarbonate (PC), Polyethylene naphthalate (PEN) and Liquid Crystal Polymer (LCP), as per the ink data sheet. The silver ink was diluted with 20% of deionized water and stirred for 10 min at 700 rpm to ensure homogeneity before loading 1 mL into the aerosol jet printer. To achieve a homogeneous ink formulation, the silver ink was diluted with 20 vol% deionized water and magnetically stirred at 700 rpm for 10 min prior to loading 1 mL into the AJP. On the other side, it was not necessary to dilute the ink for use in the micro dispensing process with the nScrypt system, as the ink performs well without dilution.

The performance was evaluated on polyimide (PI) and 3D-molded silicone substrates, chosen for their flexibility and widespread use in electronic and medical applications. Prior to printing, the substrates were cleaned with deionized water and isopropanol, followed by drying with nitrogen gas to remove any contaminants. Test patterns of 10 mm × 1 mm lines were printed and characterized on both substrates. All printed samples were dried in a convection oven at 150 °C for 30 min. The micro and nano structural properties of the printed silver layers were examined using 3D microscope (DSX1000 Olympus, Evident Corporation, Tokyo, Japan) and Scanning Electron Microscopy (SEM) (JCM-6000, JEOL Ltd., Tokyo, Japan). The thickness of the printed silver layers was measured using a profilometer (Dektak XT, Bruker Nano Surfaces GmbH, Karlsruhe, Germany). Multiple measurements were taken across the substrate to ensure uniformity, and the average thickness was reported. Electrical properties of the printed silver layers were determined using a four-point probe system (Pro4-4050, Signatone Corporation, Gilroy, CA, USA) and Keithley source meter. Measurements were taken at various locations on the printed patterns to assess uniformity and conductivity.

## 3. Results and Discussion

### 3.1. Morphology of the Printed Layer

**Figure 2:**
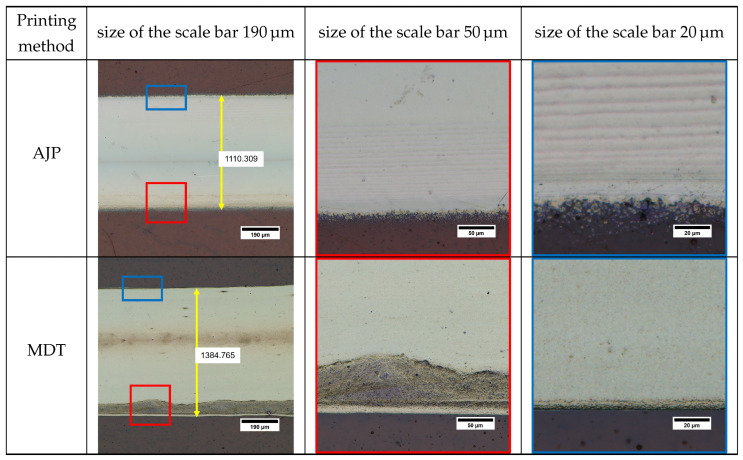
Comparison of silver-printed lines between AJP and MDT.

Figure 2 illustrates the continuous line-by-line deposition approach employed by Aerosol Jet Printing (AJP) and Micro Dispensing Technology (MDT). While both techniques employ continuous printing methods, their underlying mechanisms differ significantly, as discussed in Section 1. In Aerosol Jet Printing (AJP), an electromagnetic shutter plays a crucial role in modulating the continuous flow of aerosol mist, segmenting it into precise quantities for deposition. The resulting pattern size on the substrate is primarily influenced by the aerosol nozzle diameter, sheath and carrier gas flow rate, printing speed, and the interaction between the mist and the substrate surface. This enables AJP to achieve fine, uniform patterns with high precision. In contrast, MDT employs a fundamentally different approach to deposition. A SmartPump™ system regulates the continuous flow of ink, segmenting it into controlled droplet volumes for deposition. The resulting printing layer size is governed by factors such as the nozzle diameter, valve opening time, applied pressure, and the standoff distance between the nozzle and the substrate. This method allows MDT to achieve high-precision deposition, particularly for applications requiring thicker layers or higher material viscosity. The timing of the valve’s opening and closing regulates the material flow, influencing the line’s start and end points as well as overall continuity. MDT-printed lines exhibited sharper edges compared to AJP, but blobs were occasionally observed at the edges due to higher operating pressure [27].

Figure 2 shows that the line patterns deposited using the two approaches have similar dimensions, despite a significant difference in nozzle diameter. In AJP, a nozzle diameter of selected 300 µm was selected in conjunction with the atomizer and sheath gas flow rates to ensure stable aerosol generation and focused deposition. For low-viscosity silver nanoparticle inks, the precise control of aerosol or droplet formation is crucial to achieve high-resolution features, uniform deposition, and process stability [6,36]. An imbalance in these parameters can result in overspray, discontinuous lines, or material accumulation [36]. However, the layers printed by AJP exhibit finer “sprinkle-like” features along the edges of the lines. This phenomenon can be attributed to the nature of the aerosol mist used in AJP, which consists of extremely small ink droplets [37]. The final shape of the deposited pattern is influenced by the consistency of the aerosol mist, the focusing ratio between the sheath and carrier gas flows, and the shutter speed. The sprinkle-like spots observed along the edges result from atomized ink droplets that are not fully captured by the main focused stream of transport gas. These separated droplets create a subtle overspray pattern adjacent to the edges of the printed lines. Previous studies by Mahajan et al. [6] and Smith et al. [30] highlighted this effect, noting that the focusing ratio significantly impacts the line quality and the extent of overspray. The sprinkle spots were primarily located at the leading and trailing edges of the printed lines, corresponding to the movement direction of the deposition head relative to the substrate. Including these sprinkle features, the average width of the AJP-printed line along the deposition path was measured to be 1104.3 ± 56 μm.

Similarly, in MDT, achieving stable droplet formation at such low viscosities requires careful adjustment of dispensing pressure and the timing of valve (valve open and close timing) to avoid dripping or satellite droplet formation [27]. In contrast to the lines of AJP, the MDT exhibit a homogenous shape with the excellent edge sharpness, free from any sprinkles or extra droplets, as shown in Figure 2. The MDT printed lines have an average width of 1382.10 ± 17 μm, which is slightly larger than the lines printed by AJP. However, unlike AJP, where sprinkles are considered part of the line width, the larger feature size of MDT lines can be attributed to the ink viscosity and is finely controlled by optimizing the dispensing pressure. The smaller variation in MDT line dimensions reflects its ability to achieve a lower drop volume and greater precision during deposition. This consistency underscores the high level of control and accuracy provided by MDT, making it an effective technology for requiring uniform and sharp-edged printed patterns. While AJP is a well-established non-contact deposition technique due to its aerodynamic focusing mechanism and relatively large standoff distances (typically in millimeters) [38], MDT operates with a much smaller nozzle–substrate gap, typically in the range of 25–100 μm. Despite this proximity, the MDT process avoids direct mechanical contact between the nozzle tip and substrate. The stability of the ink bridge during dispensing is governed by capillary forces and viscous resistance within the fluid, which together enable a continuous flow of material while maintaining a physical separation between the solid surfaces. These dynamics form the basis for classifying MDT as a non-contact process. The small gap is also important for minimizing mechanical disturbance, maintaining precise droplet placement, and avoiding nozzle wear or substrate damage during high-precision printing. Temperature control is another crucial factor, particularly when printing on flexible substrates such as polyimide. Substrate temperatures of 60 °C were employed to promote controlled solvent evaporation and improve ink adhesion. However, at reduced substrate temperatures, the evaporation rate of the solvent decreases, which can lead to excessive spreading of the ink and reduced resolution of printed features [36]. This slower drying may also enhance the coffee-ring effect, wherein nanoparticles move to the edges of the printed line during solvent evaporation, resulting in non-uniform thickness and poor line definition [7]. Additionally, insufficient thermal energy at the substrate surface may compromise ink–substrate interactions, leading to weaker adhesion and potentially reduced mechanical stability of the printed structures [36].

**Figure 3:**
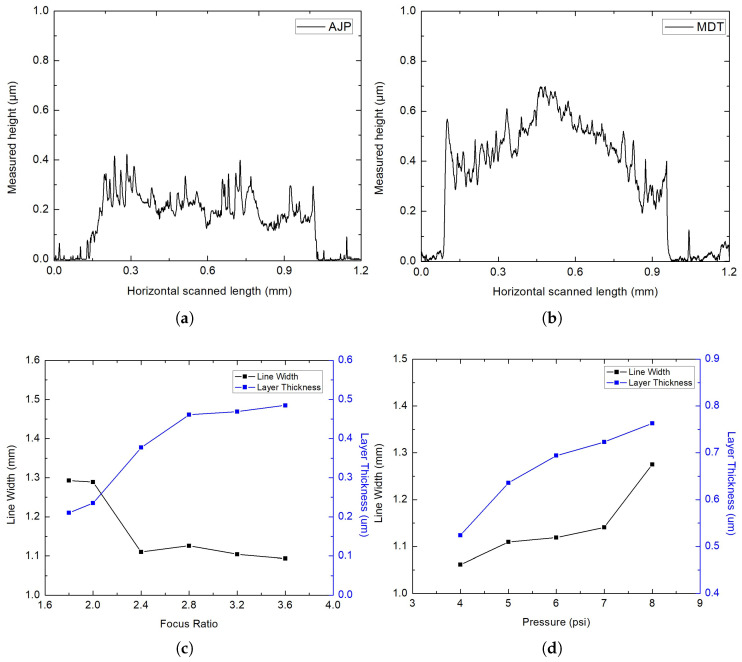
Profilometry of (**a**) AJP and (**b**) MDT; (**c**) the influences of focus ratio on line width and layer thickness in AJP; and (**d**) the effect of pressure on line width and layer thickness in MDT.

The morphologies of the deposited lines were analyzed using profilometry, and the corresponding 2D cross-sectional profiles are presented in Figure 3. Despite differences in process mechanisms and nozzle diameters, the resulting deposits display similar morphological characteristics. The width, shape, and thickness of the lines are comparable for both techniques, suggesting that similar evaporation dynamics are at play during the deposition processes for both AJP and MDT. In comparison, AJP deposits a smaller amount of ink per unit area at a focus ratio of 3.8 and a printing speed of 5 mm/s. The AJP-printed layer exhibited an average minimum height of 0.264 μm and a maximum height of 0.425 μm. However, the roughness of the AJP layer was higher (Ra = 0.11 μm) than that of the MDT layer (Ra = 0.08 μm). In the case of MDT, the deposited layer was well-printed and homogeneous, though nanoparticles tended to accumulate at the edges of the printed line. The MDT-printed layer demonstrated an average height of 0.526 μm, with a peak height of 0.698 μm. Despite these differences, the overall material deposition between the two techniques is equivalent, as shown in Figure 3. When comparing the two techniques, MDT achieved higher edge sharpness and overall pattern uniformity. AJP, on the other hand, produced thinner and less wavy layers. The deposition rate of the material in MDT depends on parameters such as applied pressure, valve opening, printing speed, and the drop distance. For AJP, the deposition is influenced by the focus ratio, printing speed, and standoff distance. Lastly, AJP and MDT each demonstrate unique characteristics, with specific process parameters determining the final printing morphology and material deposition influenced by the fill parameters.

Figure 3 shows the influence of the focus ratio on the line width and layer thickness for AJP and the effect of applied pressure for MDT. In AJP, an increase in the focus ratio, defined as the ratio of sheath gas flow to aerosol carrier flow, results in a decrease in line width while simultaneously causing a steady increase in layer thickness. This behavior indicates that higher focus ratios enhance the vertical concentration of the aerosol mist, leading to thicker deposited layers. A higher focus ratio leads to a more concentrated deposition stream, resulting in narrowing line width and increased layer thickness. For instance, at a focus ratio of 2, the line width achieved was 1.28 μm, whereas at a focus ratio of 3.8, the line width decreased to 1.09 μm, with a corresponding layer thickness of 0.485 μm. However, increasing the deposition rate per unit area significantly impacted layer formation on the substrate, leading to the observation of sprinkles.

For MDT, the applied pressure directly affects the volume of ink dispensed through the nozzle, which in turn determines the printed line width and layer thickness. As the pressure was increased from 4 psi to 8 psi, both the line width and layer thickness increased. The line width increased gradually, with a more pronounced rise at higher pressures, attributed to the higher volume of ink ejected, which spread laterally on the substrate. The line width at a pressure of 4 psi was measured as 1.06 μm, while at a pressure of 8 psi, it increased to 1.57 μm. Similarly, the layer thickness increased consistently, reflecting higher vertical deposition of material at higher pressures; at 8 psi, the measured thickness was 0.763 μm.

### 3.2. Electrical Characterization

**Figure 4:**
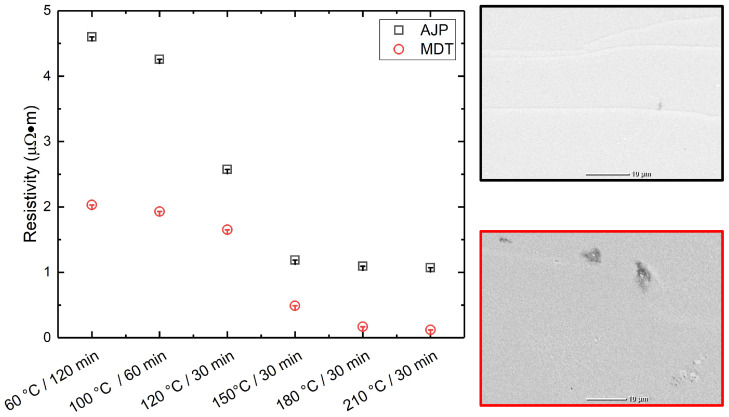
Difference in line resistivity as a function of curing temperature and time.

The average line resistivity was measured under varying post-treatment temperatures and durations using a four-point probe method using the Signatone Pro4-4050. Overall, a trend of decreasing resistivity with increasing curing temperature and duration was observed, indicating improved sintering and interparticle connectivity. For AJP-printed samples, the lowest resistivity was recorded at 210 °C for 30 min, yielding 1.07184 μΩm, while for MDT-printed lines, the minimum resistivity achieved under the same curing condition was significantly lower at 0.12098 μΩm. This trend correlates with the changes in structural morphology observed with different post-treatment conditions, as shown in Figure 4. Lower resistivity values were observed in denser silver layers formed at higher hot convection oven temperatures. In the case of AJP, the resistivity of the silver lines is more sensitive to curing temperature and duration, highlighting its reliance on precise sintering conditions. The thinner cross-sectional profile of AJP lines may also contribute to higher resistivity. Between the two techniques, MDT consistently yielded lower resistivity across all curing conditions. This advantage is likely due to its ability to deposit thicker, more uniform lines with improved particle packing and distribution, resulting in enhanced electrical performance.

### 3.3. 3D Layer Morphology on Silicone

The pattern design process involves defining shape geometries, including start and end positions, through numerical calculation. These definitions determine the transfer path, offering greater flexibility in vector-based processing compared to the traditional raster-based, line-by-line approach. In AJP, the printing path involves opening the shutter, enabling the aerosol mist to be directed onto the substrate. During non-printing transfer paths, the shutter remains closed, preventing mist deposition. For MDT, the ink flow is controlled by a valve mechanism. When the valve is open, the ink flows through the nozzle and is deposited onto the substrate. To stop dispensing, the valve rod shifts into a closed position during non-printing transfer paths. For more complex designs or patterns, fill algorithms are employed. These algorithms control the movement of the substrate beneath the deposition head in a predefined manner, enabling precise pattern printing in the X-Y plane and, when necessary, conformally along the Z-axis.

**Figure 5:**
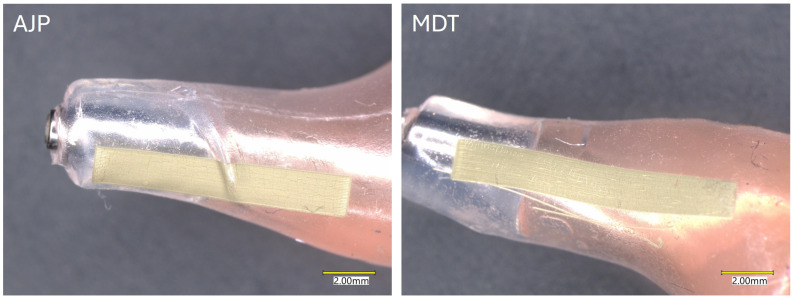
Printing on 3D mold silicone substrate through AJP and MDT.

For both printing processes, the same printing parameters, as outlined in Table 1, were utilized. However, the standoff distance varied to account for the changing 3D height of the substrate. Silver lines printed using AJP and MDT on 3D substrates exhibited distinct morphological features compared to those printed on flat surfaces. The curvature and surface topography of the substrate significantly affect the uniformity, continuity, and edge definition of the printed lines in both processes as shown in Figure 5. On highly curved surfaces, AJP lines frequently exhibited sprinkle-like artifacts near the edges, likely resulting from incomplete aerosol focusing and variations in ink atomization. The standoff distance—the gap between the nozzle tip and the substrate—was identified as a critical parameter for ensuring consistent material deposition. On highly curved surfaces, variations in standoff distance caused by surface irregularities led to uneven material deposition, resulting in fluctuations in line width, thickness, and edge roughness. Achieving continuous and homogeneous deposition with AJP requires precise control of the fill algorithm and path planning. A well-optimized algorithm minimizes overlaps and gaps during printing, which is especially important on uneven surfaces. Additionally, adjustments to the printing path—such as varying the nozzle orientation—enable accurate material deposition across substrates with complex geometries.

In MDT, silver lines printed on 3D surfaces showed minor variations in width and edge sharpness as the substrate’s curvature or slope increased. Maintaining a consistent standoff distance was crucial for ensuring uniform material deposition. However, when the surface exhibited excessive slope or curvature, deviations in the standoff distance resulted in slight irregularities in line morphology. Unlike AJP, MDT relies on controlled pneumatic pressure and valve timing for material flow. These parameters contributed to the consistent cross-sectional profiles of printed lines on most 3D surfaces, although edge sharpness could be slightly reduced in highly contoured areas. Hence, crack formation was easily observed in the cured samples from both printing processes after oven treatment.

On PI substrates, AJP produced lines with excellent adhesion, exhibiting strong bonding and passing the tape test without any signs of delamination. AJP also performed well on 3D silicone surfaces, with printed lines maintaining good adhesion even under mechanical bending or stress. This robust performance is likely attributed to the fine aerosolized droplets, which enable the ink to conform more effectively to the surface texture of the silicone. In contrast, adhesion on PI was also strong for MDT lines, with no significant delamination observed during testing. However, on silicone, MDT lines showed weaker adhesion compared to AJP, especially on 3D surfaces. The thicker lines deposited by MDT had less flexibility, and some delamination was observed when subjected to mechanical strain. This may be due to the ink’s inability to fully penetrate the surface of the silicone, combined with the thicker material’s reduced ability to conform to the surface geometry.

Table 2 shows a comparison between two leading printed electronic processes AJP and MDT.

In addition to layer morphology, different fabrication technique and economic parameters were compared. Ink flexibility is a limiting factor in AJP compared to MDT, as it can only accommodate low-viscosity inks ranging up to 1000 cP with small particle sizes. In contrast, MDT can process inks with much higher viscosities, as well as larger particle sizes and higher material loadings. This flexibility provides a significant advantage for MDT, as it enables the use of a wider range of printable materials. The relationship between printing process parameters and layer thickness in both AJP and MDT is strongly influenced by the material properties of the ink being used. The MDT process relies on the continuous dispensing of materials, which inherently allows for higher printing speeds compared to the atomization-based deposition in AJP. Mass production is another critical consideration. In MDT, the integration of multiple nozzles in an array for large-area deposition is well-developed. A direct cost comparison between AJP and MDT is difficult to establish due to the variability in system configurations, material requirements, and operational conditions.

**Figure 6:**
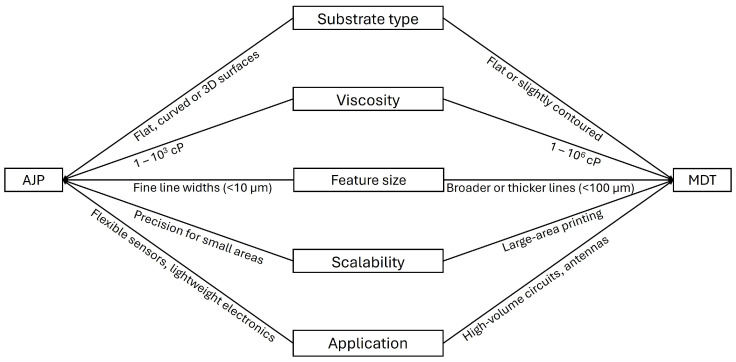
Flowchart for selecting the printing process: AJP and MDT.

One of the primary challenges associated with AJP and MDT technologies is the need to understand, predict, and optimize the numerous parameters that influence the print quality of the deposited ink material. Several factors determine the effectiveness of selecting the most suitable printing technique, as illustrated in the flowchart in Figure 6. Among these factors, material properties and ink formulation represent the most critical challenges for achieving successful fabrication of 2D- and 3D-printed electronics using AJP and MDT.

## 4. Conclusions

A detailed comparison of the layer morphologies fabricated using AJP and MDT was conducted. Both continuous printing techniques were used to fabricate and evaluate line patterns. Despite the significant differences in nozzle diameters used in AJP and MDT, the resulting lines exhibited similar overall morphologies. However, lines printed with AJP displayed sprinkle-like artifacts along the edges, whereas MDT produced lines with sharply defined edges and superior layer uniformity. AJP demonstrated the capability to deposit thinner layers and narrower line widths compared to MDT. In contrast, MDT allowed for the fabrication of a wider range of layer thicknesses and line widths, offering greater versatility for diverse application needs. The morphology of lines printed using AJP was primarily influenced by the focus ratio and printing speed. Additionally, the roughness of AJP layers was strongly affected by the fill algorithm applied during the deposition process. For MDT, the material deposition was predominantly governed by pneumatic pressure, standoff distance, and printing speed. The morphology of MDT-printed lines was influenced by the timing of valve opening and closing, which governs ink flow dynamics in relation to nozzle diameter. Additionally, the electrical resistance of printed lines in both techniques was found to depend on the temperature conditions during post-processing, underscoring the critical interplay between thermal treatment and the resulting structural properties. While MDT demonstrated superior 3D conformality compared to AJP, quantitative analysis of line width variation, thickness uniformity, and edge roughness on curved 3D surfaces remains a significant technical challenge. The complex geometries and limited accessibility of 3D substrates limit the application of standard measurement techniques such as profilometry, optical microscopy, or SEM, which are typically optimized for planar surfaces. According to these observations, AJP-printed lines exhibited noticeable width variation and instability on sloped or curved regions, while MDT maintained consistent feature dimensions, indicating better material control and adhesion on 3D surfaces. The versatility and scalability of MDT, combined with its ability to process a wider range of materials, make it a favorable choice for printed and flexible electronic applications requiring high throughput and large-area printing. Ultimately, both techniques offer distinct advantages and are well suited for the fabrication of advanced microelectronic devices and sensors.

## Figures and Tables

**Table 1:** Printing parameters for morphological study of silver ink.

Printing Parameters	AJP	MDT
Nozzle diameter (μm)	300	50 I.D/100 O.D
Printing speed (mm/s)	5	40
Sheath flow (sccm)	50–60	-
Atomizer flow (sccm)	25–35	-
Atomizer pwr	45–48	-
Valve opening (mm)	-	3.190
Valve closing (mm)	-	3.170
Dispensing pressure (psi)	-	4
Valve operating speed (mm/s)	-	0.099
Substrate temperature (°C)	60	60
Substrate standoff distance (mm)	4	0.2
Thermal curing (°C, min)	150, 30	150, 30

**Table 2:** Comparison between AJP and MDT printing.

Characteristic	AJP (Optomec, Aerosol Jet^®^ 300)	MDT (nScrypt, 3Dn-300)
Deposition Method	Uses an atomized aerosol stream focused by a sheath gas, allowing precise and uniform deposition [30].	Relies on positive displacement and precise valve control to dispense material directly from the nozzle [39].
Pressure Requirements	Utilizes low-pressure aerosol streams for atomization and deposition [30].	Operates under higher pressures (e.g., 1–100 psi) to drive material flow through the nozzle [27].
Resolution	Achieves fine features with a line width as small as 10 µm, ideal for high-resolution printing [4,40].	Line widths typically start at 50–100 µm, depending on nozzle diameter, offering precision for larger-scale features [8].
Material Compatibility	Handles a wide range of materials, including nanoparticle inks, polymers, and biological materials. Supports low-viscosity solutions effectively [41].	Compatible with high-viscosity materials like pastes, adhesives, and conductive inks, enabling the deposition of denser materials [39].
Substrate Versatility	Prints on flat, curved, or 3D surfaces with limited conformality, making it suitable for non-planar applications [30,42].	Primarily suited for flat or slightly contoured substrates. Non-contact capability reduces damage risk and highly convenient for 3D surfaces [23,43].
Speed	Relatively slower deposition rates due to its fine-resolution process.	Faster printing speeds, particularly for high-viscosity materials, allowing for quicker deposition.
Layer Thickness Control	Capable of depositing thin layers with nanometer-scale precision; ideal for multilayer structures [6,8].	Delivers thicker layers, suitable for applications where structural or conductive thickness is critical [23].
Material Waste	Minimal waste due to precise aerosol focus and efficient material usage.	Slightly higher waste compared to AJP, as some material may remain unused in the nozzle or system.
Ease of Maintenance	Requires frequent cleaning of the atomizer and aerosol path to avoid clogging, especially with nanoparticle inks.	Easier to maintain with fewer clogging risks, especially when handling viscous materials.
Cost of Operation	Higher operational costs due to atomization systems and precise gas controls.	Lower operational costs due to simpler material flow control mechanisms.

## Data Availability

The data that support the findings of this study are available from the corresponding author upon reasonable request.
